# [Retracted] Notch1 induces epithelial-mesenchymal transition and the cancer stem cell phenotype in breast cancer cells and STAT3 plays a key role

**DOI:** 10.3892/ijo.2025.5752

**Published:** 2025-05-12

**Authors:** Xiaojin Zhang, Xiaoai Zhao, Shan Shao, Xiaoxiao Zuo, Qian Ning, Minna Luo, Shanzhi Gu, Xinhan Zhao

Int J Oncol 46: 1141-1148, 2015; DOI: 10.3892/ijo.2014.2809

Following the publication of this paper, it was drawn to the Editor's attention by a concerned reader that, in Fig. 3A and B on p. 1145 showing the results of cell invasion and migration experiments, four pairs of overlapping data panels were identified, affecting half the panels shown in the figure, such that these data panels, which were intended to show the results of differently performed experiments, had all apparently been derived from the same original source.

Owing to the large number of duplications of data that have been identified in this paper, the Editor of *International Journal of Oncology* has decided that it should be retracted from the Journal on account of a lack of confidence in the presented data. The authors were asked for an explanation to account for these concerns, but the Editorial Office did not receive a reply. The Editor apologizes to the readership for any inconvenience caused.

